# Supplementary material for: Architecture and flexibility of native kinetochores revealed by structural studies utilizing a thermophilic yeast
Source: bioRxiv. 2024 Feb 29:2024.02.28.582571. Preprint. [Version 1] doi: 10.1101/2024.02.28.582571 (PMC10925344; doi:10.1101/2024.02.28.582571)
Supplement: Supplement 5 [file NIHPP2024.02.28.582571v1-supplement-5.pdf]

bioRxiv preprint doi: https://doi.org/10.1101/2024.02.28.582571; this version posted March 1, 2024. The copyright holder for this preprint (which was not certified by peer review) is the author/funder, who has granted bioRxiv a license to display the preprint in perpetuity. It is made available under aCC-BY 4.0 International license.

Supplementary Materials

Supplementary figures 1 to 4

Supplementary movies 1 to 4

Supplemental Movie 1. A movie scanning through the Z axis of a reconstructed tomogram containing a singlet kinetochore. The movie was taken at 25 frames per second and the scale bar is 100nm.

Supplemental Movie 2. A movie scanning through the Z axis of a reconstructed tomogram containing a doublet kinetochore. The movie was taken at 25 frames per second and the scale bar is 100nm.

Supplemental Movie 3. Dynamics of an individual kinetochore with conformational changes observed in the handle regions over time. The scanning rate ~1 s/frame. The x-y scale bar is 50 nm.

Supplemental Movie 4. Dynamics of an individual kinetochore highlighting changes observed in the brush head over time. The scanning rate ~1 s/frame in. The x-y scale bar is 50 nm.

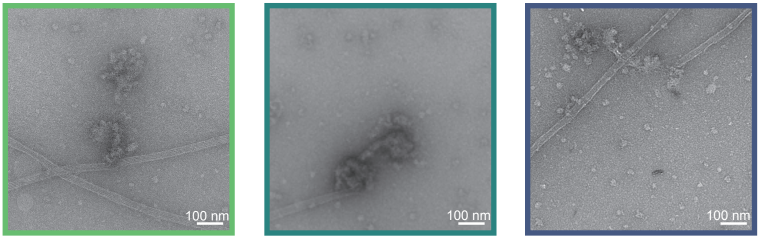

Supplemental Figure 1. Representative images of kinetochores interacting with microtubules on either the lattice (left), tip (center), or both (right). Scale bars are 100 nm.

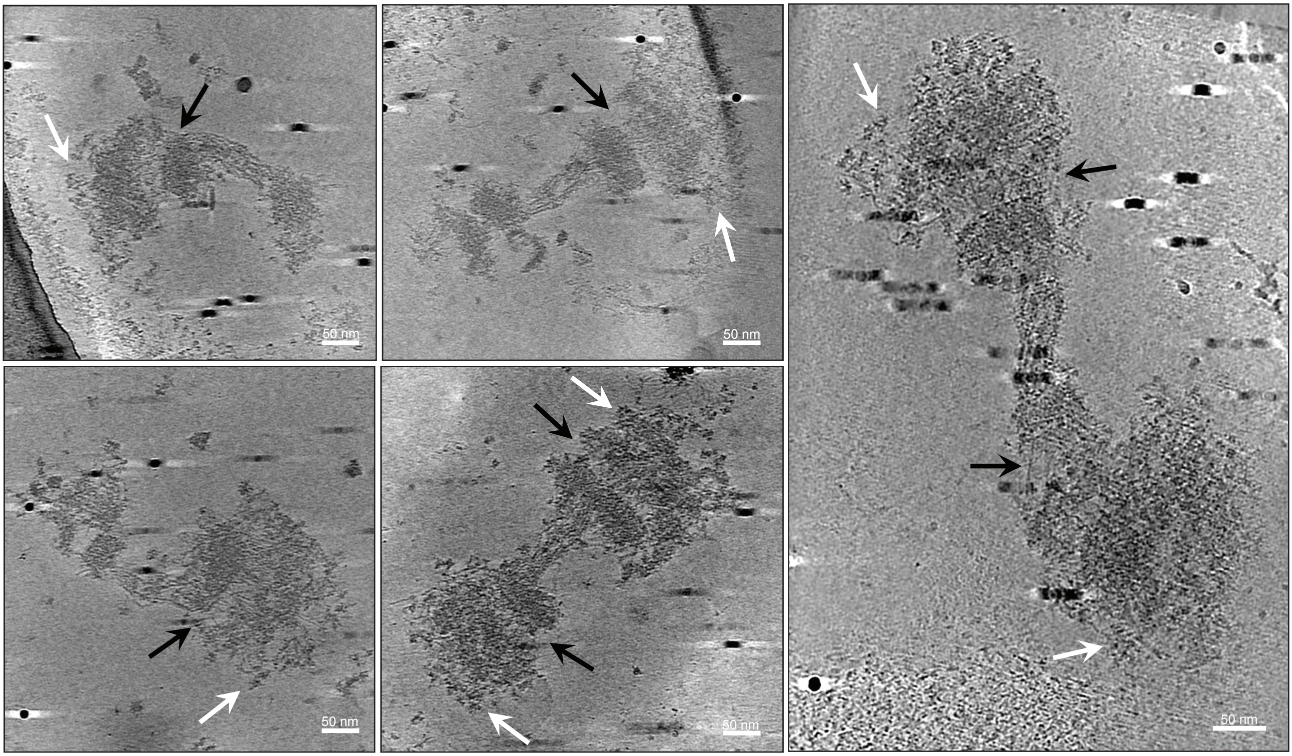

Supplemental Figure 2. Gallery of kinetochores revealed by cryo-electron tomography. Black arrows: flexible fibrils connecting the brush tips to the compact hub. White arrows: brush tip extensions. Scale bars are 50 nm in each panel.

bioRxiv preprint doi: https://doi.org/10.1101/2024.02.28.582571; this version posted March 1, 2024. The copyright holder for this preprint (which was not certified by peer review) is the author/funder, who has granted bioRxiv a license to display the preprint in perpetuity. It is made available under aCC-BY 4.0 International license.

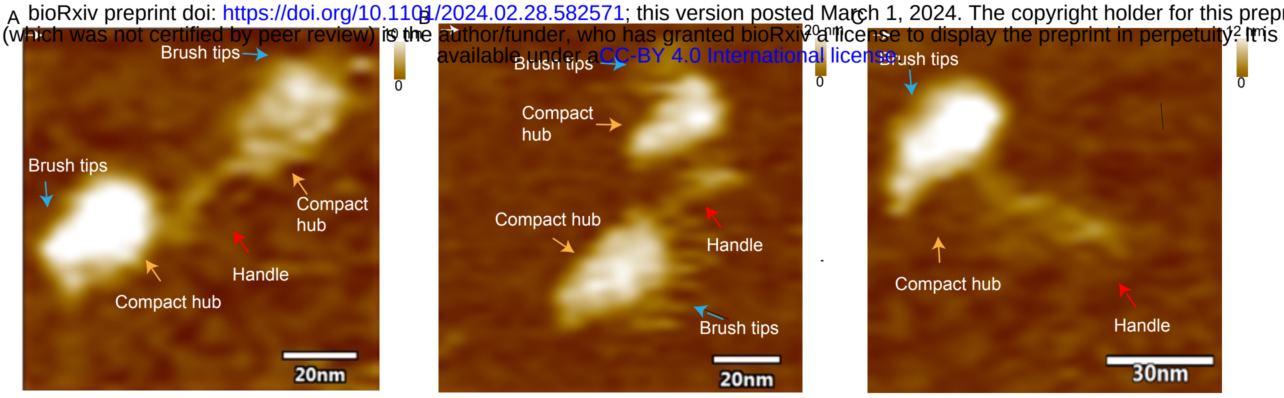

Supplemental Figure 3. Three examples of AFM height images of double kinetochores. The blue arrows indicates the brush tips, the orange arrows indicates the compact hub, and the red arrow indicates the brush handle. The x-y scale bar is 20 nm. The scanning rate is 1 s/frame with 256 x 256 pixels. White arrows at the top left of the images indicate the scanning direction. The z-scale is 0 to 20 nm (dark to light brown).

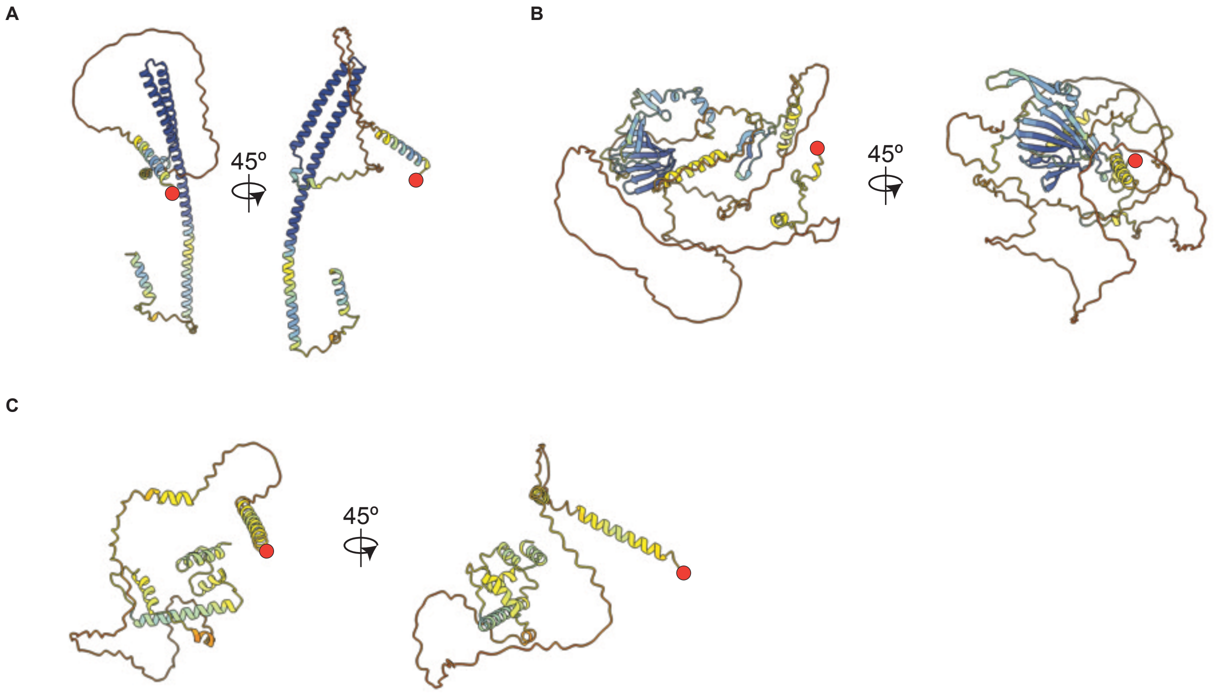

Supplemental Figure 4. Alphafold ColabFold predictions of Ame1, Mif2, and Cnn1 show long disordered tails. A) A structural prediction for *K. marxianus* Ame1 with its N-terminus marked with a red circle. B) A structural prediction for *K. marxianus* Mif2 with its N-terminus marked with a red circle. C) A structural prediction for the potential *K. marxianus* Cnn1 homolog with its N-terminus marked with a red circle. All models are colored by per-residue confidence score (pLDDT) where blue indicates high confidence and red indicates low confidence.
